# Split Notochord Syndrome with Spinal Column Duplication and Spinal Cord Lipoma: A Case Report

**DOI:** 10.3390/children9081138

**Published:** 2022-07-29

**Authors:** Fayez Alelyani, Keith Aronyk, Hashim Alghamdi, Ibrahim Alnaami

**Affiliations:** 1 Department of Neurosurgery, King Khalid University Medical City, King Khalid University, Abha 61413, Saudi Arabia; m.almakhfor@kku.edu.sa; 2 Department of Neuroscience, University of Alberta, Edmonton, AB T6G 2B7, Canada; keith.aronyk@albertahealthservices.ca; 3 Department of Pediatric Surgery, Abha Maternity and Children Hospital, Abha 62562, Saudi Arabia; haalghamdi2@moh.gov.sa; 4 Division of Neurosurgery, Department of Surgery, College of Medicine, King Khalid University, Abha 62523, Saudi Arabia; 5 Department of Pediatric Neurosurgery, Abha Maternity and Children Hospital, Abha 62562, Saudi Arabia; 6 Department of Neurosurgery, Aseer Central Hospital, Abha 62523, Saudi Arabia

**Keywords:** diastematomyelia, lipomyelomeningocele, split cord malformation, split notochord syndrome

## Abstract

Background and Importance: Split notochord syndrome (SNS) is an exceedingly rare type of spinal dysraphism. SNS is sometimes associated with other congenital dysraphic defects but, as in our case, the association with spinal cord lipoma, tethered cord, and spinal deformity in the form of spinal column duplication would be exceedingly rare. Herein, the authors report a three-year-old child presented with SNS associated with complex spinal deformity and other associated congenital anomalies. The patient underwent microsurgical release of the tethering element with excellent short- and long-term outcomes. Clinical Presentation: A male newborn with healthy nonconsanguineous parents was born with multiple gastrointestinal and genitourinary anomalies, and duplicated vertebral columns at the lumbosacral area consistent with split notochord syndrome. The patient was initially managed for the gastrointestinal and genitourinary anomalies. As there was no obvious neurological deficit initially, the neurosurgical intervention was postponed till the child reached 30 months of age, when he underwent uneventful release of both spinal cords at their spit point. Conclusions: SNS is an exceedingly rare developmental anomaly that is usually associated with varying degrees of complex congenital dysraphic defects. Early clinical diagnosis, understanding of the pathophysiology of spinal cord tethering, and microsurgical cord untethering are the important steps in optimal management.

## 1. Background and Importance

Split notochord syndrome (SNS) is a congenital anomaly of the central nervous system (CNS) which is believed to be a result of intrauterine insult to the growing neural tube, notochord, and mesoderm by unknown cause [1]. As a result, SNS usually involves CNS and multi-organ systems. The defect is thought to occur during the gastrulation stage in the third week of embryogenesis likely due to a persistent neurenteric canal. However, the antenatal diagnosis of any split cord malformations would be challenging due to the low resolution of ultrasonography compared with magnetic resonance imaging or computed tomography. Sometimes, the diagnosis is even made much later in the adolescent or adult age range when the patient presents with back pain and/or radiculopathy [2,3]. The mainstay of treatment of SNS is surgical exploration and spinal cord untethering in an attempt to preserve neurological function and prevent clinical deterioration, even in asymptomatic patients, although this is still debated by some [4].

## 2. Clinical Presentation

A male newborn with healthy nonconsanguineous parents was delivered through normal vaginal delivery at Abha maternity and children’s hospital. The baby was found to have an imperforate anus and recto-urethral fistula and was noticed to have a soft skin-covered midline swelling at the lumbosacral area with no other cutaneous manifestations; however, the baby was moving both lower limbs with no obvious anomaly or neurological deficit.

His abdominal imaging revealed a small-sized right kidney that is seen in the right pelvis region toward the midline posterior to the bladder, and a hypertrophied left kidney with moderate hydronephrosis and posterior urethral valve.

From a pediatric surgery perspective, the patient underwent staged repair for the imperforate anus in the form of an emergency colostomy at the start, followed by PSARP (posterior sagittal ano-rectoplasty) with division of the recto-urethral fistula, and after a few months, the patient underwent a reversal of the colostomy. These procedures went well with no complications. During this course, pediatric neurosurgery team followed the patient closely in the clinic, observing the neurological development and mainly the movement in the lower extremities.

The child started to walk with support at 14 months and was able to walk with no support at 18 months. Magnetic resonance imaging(MRI) of the spine was obtained twice through the course of follow-up.

**Figure 1:**
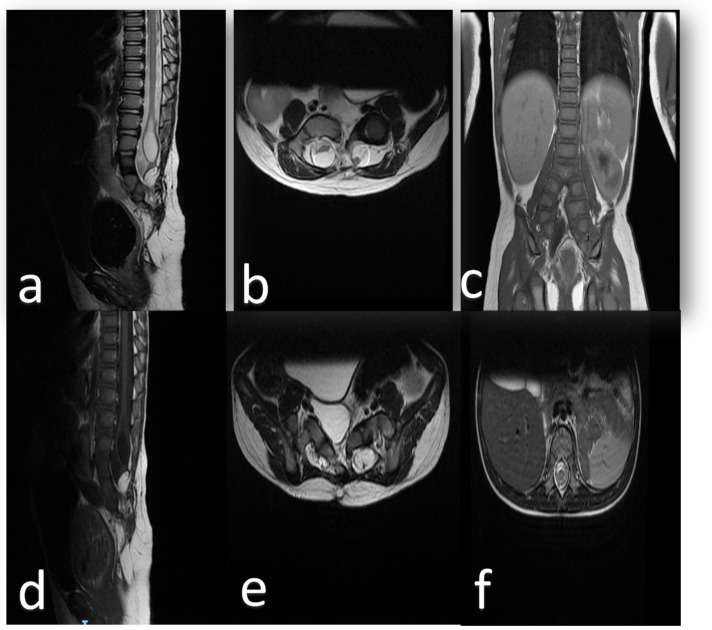
MRI (**a**) T2 sequence in the sagittal view shows hyperintense intramedullary syrinx associated with cord splitting at multiple lumbar segments. (**b**) Axial view shows the duplicated dural sacs. (**c**) Coronal view illustrates the associated spinal column division at the level of the aortic bifurcation. (**d**) T1 sequence in the sagittal view shows the same findings in (**a**); however, the lipoma is demonstrated caudally. (**e**) T2 sequence in the axial view the 2 duplicated cords inferior to the bifurcation level. (**f**) T2 sequence in the axial view the one spinal cord superior to the bifurcation level.

**Figure 2:**
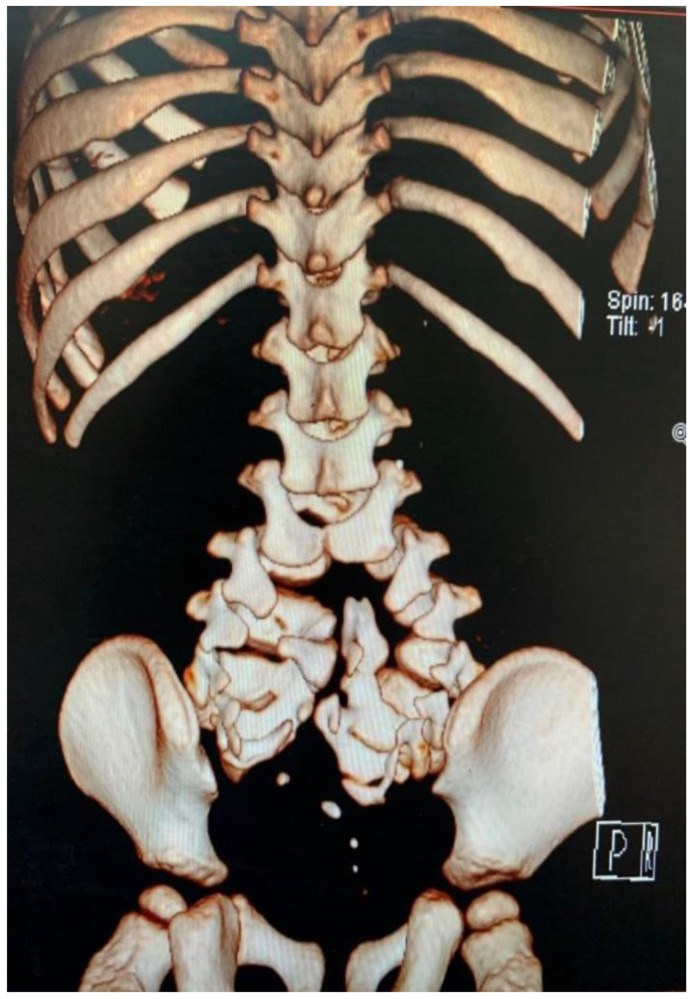
Three-dimensional reconstruction of computed tomography of the spine from mid-thoracic region to sacrum reveals duplication of the vertebral column from lumbar vertebrae 3 to the coccyx.

The MRI scan revealed a split cord at the junction level of the first and second lumbar vertebral bodies L1/L2 associated with a low-lying, tethered cord. There was a complex low T1 sequence, high T2 sequence signals, and a non-enhancing heterogeneous intensity lesion representing a lipoma extending from the posterior elements of the thecal sac into the interspinous process area in association with a huge syrinx at the terminal spinal cord. Both divided spinal cords were ending as cauda equina segments. The bony spine was also abnormal in the form of a duplicated spinal column over multiple segments beginning at the L3 level. (Figure 1 and Figure 2).

At the age of 30 months, the parents noticed more frequent falls. Clinical examination revealed mild spasticity in the right lower limbs with hyperreflexia for both knee and ankle reflexes, more on the right side than the left; however, there was preserved motor power and sensory function in both upper and lower limbs. Plantar responses were equivocal bilaterally and no clonus was observed.

**Figure 3:**
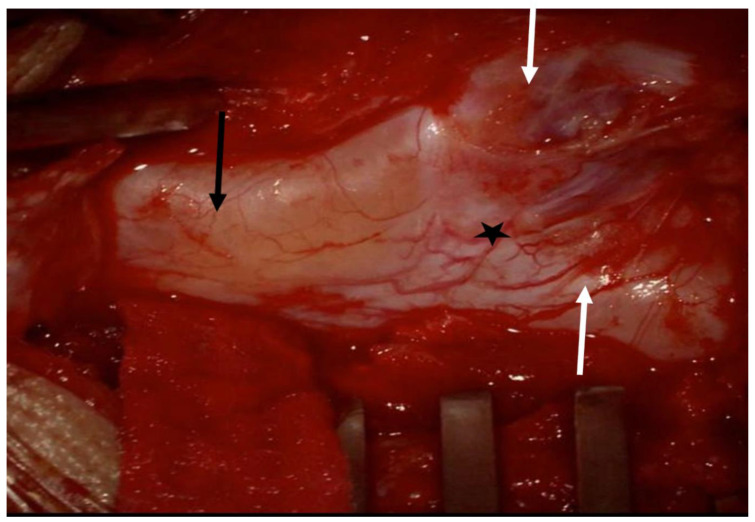
An intra-operative photo illustrates the split spinal cord within two dural sacs (SCM type II) prior to dural opening. The black arrow points to the spinal cord proximal to the bifurcation point. The stars points to the bifurcation points. The two white arrows point to the two cords distal to the bifurcation point.

**Figure 4:**
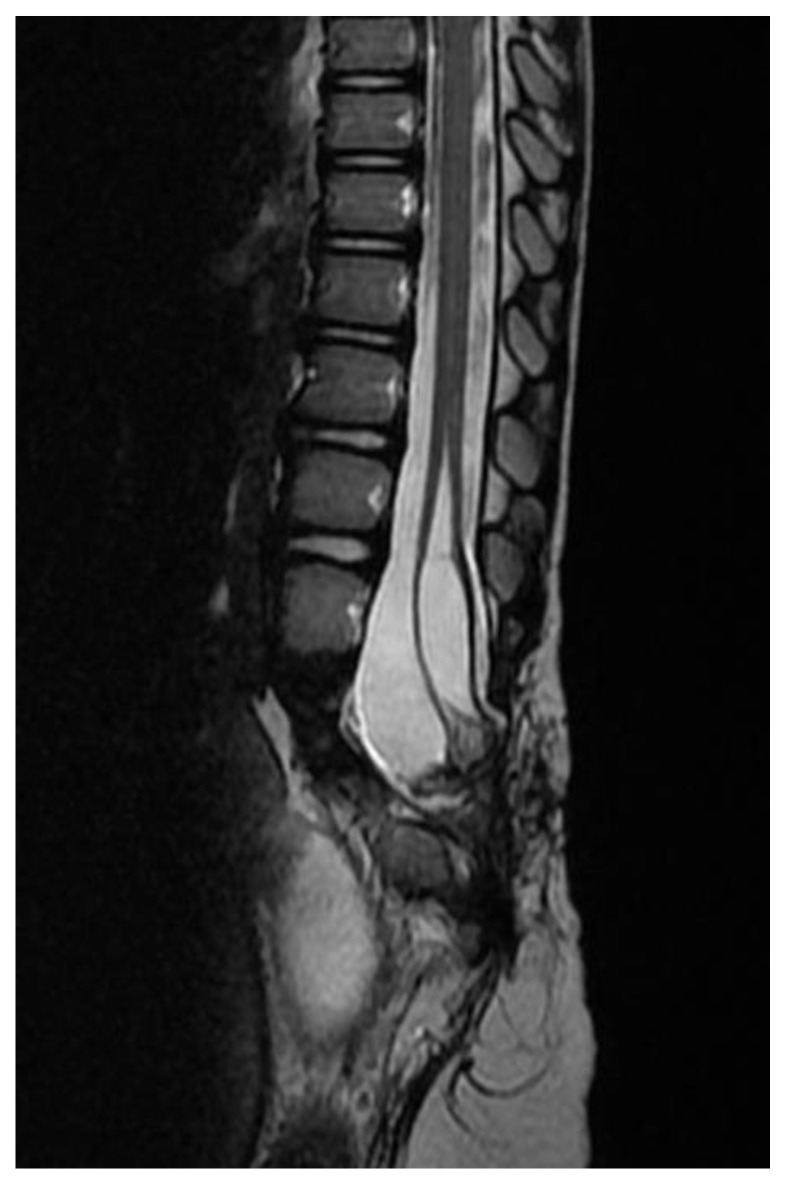
Post-operative T2 sequence in the sagittal view demonstrates a significant reduction in the size of the intramedullary syrinx.

After a prolonged discussion with the parents regarding surgery and going over the pros and cons of surgical intervention, the patient was prepared for surgery. The patient was taken to the operating room for T12-L4 laminectomy. The cord was low lying and there were separate double dural tubes (Figure 3). The dura was opened and spinal hemi-cords were explored and fully released using a microsurgical technique and neurophysiological monitoring. The dura was closed primarily. There were no complications during surgery and the intraoperative neurophysiological monitoring remained stable throughout the procedure. The patient was discharged home a few days after the surgical procedure. An MRI of the thoracolumbar spine was obtained six months after surgery and revealed a significant reduction in the size of the syrinx (Figure 4), and clinically at the one-year follow-up assessment, the patient remained neurologically stable with significant improvement of gait and resolution of the clinical signs of lower limb spasticity; however, the child is not yet potty trained and is awaiting follow-up with pediatric surgery and urology, as he needs intermittent catheterizations that are undertaken by his parents at home.

## 3. Discussion

Back in 1960, Bentley and Smith were the first researchers who reported that the abnormal splitting of the notochord may result in a wide spectrum of anomalies that could involve the vertebral column, spinal cord, and visceral organs. Split notochord syndrome is an exceptionally rare pathology, especially when accompanied by complex additional congenital anomalies. Two terms coexist interchangeably in the published literature: “split notochord syndrome” and “spine duplication syndrome” and more than 40 cases have been reported [5].

The terminology, embryogenesis, and clinical features of SNS are similar to those of split cord malformations (SCMs), the embryology and classification of which have been clarified considerably by Dias and Pang et al. The split notochord syndrome is considered to be an extreme form of SCM [6].

Pang et al., in their unified theory, proposed that the entirety of SCMs are likely to result from one basic ontogenetic error during gastrulation in the third week of embryogenesis. This error is related to a failure of resolution of the primitive neurenteric canal which is thought to eventually lead to the formation of a persisting abnormal neurenteric fistula (endomesenchymal tract) through the midline connecting the endodermal yolk sac elements with the developing neuroectodermal elements. The final pathway that would then shape the morphology depends on many factors including the healing process around the endomesenchymal tract, and the persistence or regression of the endomesenchymal tract, in addition to the final fate of the misplaced midline structures.

It has been observed that higher levels of maternal plasma homocysteine concentrations and the methionine synthase reductase (MTRR) gene polymorphism are associated with the occurrence of neural tube deformities [7]. It was also noticed that motor neuron formation is affected by excessive *Shh* sonic (hedgehog) expression, which is found to stimulate floor plate (notochord) differentiation [8] and could result in duplication of the neuraxis. The *Shh* gene is expressed in the early primitive node stage and it is postulated that it may cause a forward migration of the posterior elements of the neural tube and may play a role in the pathogenesis of the neural tube duplication and rare cases of diplomyelia or diastematomyelia [9].

Spinal duplication is one of the distinguishing features of SNS and this duplication may range from the splitting of just the sacrum and coccyx to duplication of the entire lumbar vertebrae. The development of spinal column duplication is thought to result from failure of fusion of the ossification centers located laterally of the vertebral bodies and often encountered in the dorsolumbar area [10].

Varying degrees of duplicated spinal columns and spinal cords are usually noticed in both SNS and caudal duplication syndrome. This results in a spectrum of dysraphic manifestations from a simple fibrous band that divides the spinal cord to duplication of the entire caudal structures. To diagnose caudal duplication syndrome, associated duplication of other mesodermal/caudal cell mass structures such as duplications within vascular, genitourinary, and gastrointestinal systems would be considered to exist [11].

SNS is considered to be a complex developmental anomaly related to a basic error in embryogenesis often affecting the entire caudal end of the developing embryo. Several case reports have described the associated findings involving the gastrointestinal system [12].

In our case, the spine column was involved more than the caudal cell mass derivatives. The spine was duplicated from the lumbar to sacral levels and the spinal cord divided into two hemi-cords. These morphological findings are also associated with spinal cord tethering due to the associated intramedullary lipoma.

The main neurological features of SNS are the deficits related to spinal cord abnormalities, which are often associated with tethering elements at the terminal end of the spinal cord. Spinal cord tethering is thought to lead to traction on the spinal cord, especially during rapid growth in early childhood and adolescence. The abnormal tension within the neuraxis may result in ischemic damage to the caudal neural tube structures [13,14].

The incidence of SNS associated with both intramedullary lipoma and spinal duplication is still unknown. Usually, spinal cord lipomas occur as more isolated lesions. However, there are a few case reports describing lipomyelomeningocele associated with congenital malformations such as split cord malformation (SCM) type II and other developmental anomalies [15]. One study showed a 24% association of SCM with lipomyelomeningocele [16].

A whole spine MRI with contrast would be the investigation of choice looking for associated abnormalities such as lipoma, neurenteric cyst, hydro syringomyelia, or dermoid/epidermoid cysts.

Surgical treatment for patients with SNS associated with a tethering spinal cord lipoma is usually advised in an attempt to prevent neurological deterioration, despite the patient being symptom-free, and having no neurological deficit [17].

The main goal of the surgery would be spinal cord untethering and dural tube reconstruction. The overall outcome of SNS is variable, often depending on the status of associated visceral malformations.

Our patient had a favorable early clinical outcome at the one-year post-operative stage. However, long-term clinical and radiological follow-up will be important considering the relatively high rate of late retethering [18].

Long-term follow-up for these patients is crucial to address late consequences or complications of interventions, such as retethering, hydrocephalus, and consequences of cerebrospinal fluid diversion procedures if required in addition to complications related to other symptoms such as hydronephrosis [19,20].

The usual limitation of this type of study is being an isolated case report; however, it is an exceedingly rare condition. In the presence of a wide spectrum of anomalies of spinal duplication and such varying degrees of associated visceral defects, and the rarity of the condition, it is hard to unify management strategies. More data will have to be compiled into case series to be able to begin determining optimal management.

## 4. Conclusions

SNS is a rare and complex form of spinal dysraphism, especially when it is associated with sacro-coccygeal deformities and spinal cord lipomatous tethering elements. In general, the literature is supportive of surgical treatment in an attempt to prevent neurological deterioration; the authors present this rare case of complicated split notochord syndrome in an attempt to add to the literature describing the complex anomalies associated with congenital spinal dysraphism.

## Data Availability

All data generated or analyzed during this study are included in this article. Further inquiries can be directed to the corresponding author.

## Abbreviations

CNS: Central nervous system, SCM: Split cord malformation, SNS: Split notochord syndrome, CT: Computed tomography scan, MRI: Magnetic resonance imaging.
